# Natural Antimicrobials from Plants Used as Food Preservatives

**DOI:** 10.3390/foods15081309

**Published:** 2026-04-10

**Authors:** Antia G. Pereira, Ana Perez-Vazquez, Paula Barciela, Ana O. S. Jorge, Ezgi Nur Yuksek, Miguel A. Prieto

**Affiliations:** 1 Nutrition and Food Group (NuFoG), Instituto de Agroecoloxía e Alimentación (IAA), Campus Auga, Universidade de Vigo, 32004 Ourense, Spain; ana.perez.vazquez@uvigo.es (A.P.-V.); paula.barciela@uvigo.es (P.B.); ezginur.yuksek@uvigo.es (E.N.Y.); 2 Nutrition and Food Group (NuFoG), Galicia Sur Health Research Institute (IIS Galicia Sur), SERGAS-UVIGO, 36213 Vigo, Spain; 3 REQUIMTE/LAQV, Department of Chemical Sciences, Faculty of Pharmacy, University of Porto, R. Jorge Viterbo Ferreira 228, 4050-313 Porto, Portugal

**Keywords:** phytochemicals, bioactive compounds, plant-based antimicrobials, food safety, microbial inhibition, shelf life, natural preservatives, toxicological assessment

## Abstract

Plant-derived antimicrobial compounds are emerging as promising alternatives to synthetic preservatives in the food industry due to their efficacy against a broad spectrum of pathogenic and spoilage microorganisms, as well as their consumer acceptance. This review critically examines the main classes of bioactive phytochemicals, including essential oils, polyphenols, alkaloids, terpenoids, and saponins, comparing their relative antimicrobial effectiveness and highlighting representative examples. Notably, essential oils rich in thymol or carvacrol have shown strong inhibitory activity against *Listeria monocytogenes* and *Salmonella* spp., while polyphenols and alkaloids exhibit moderate to strong activity depending on concentration and food matrix. Their mechanisms of action include cell membrane disruption, inhibition of key enzymes, and interference with DNA or protein synthesis. Applications in food systems (i.e., incorporation into coatings, emulsions, or controlled-release formulations) demonstrate potential for extending shelf life and enhancing safety. However, practical implementation is challenged by matrix-dependent efficacy, compound stability, sensory impact, and regulatory and toxicological considerations. By synthesizing current knowledge, identifying the most promising compound classes, and highlighting key limitations, this review provides a critical framework to guide future research and the development of effective, sustainable natural preservatives in the food industry.

## 1. Introduction

Food security, along with disease prevention, energy resource availability, and the management of health and environmental crises, are among the main challenges humanity has faced throughout history [1,2]. Within the dominion of food security, key challenges include nutritional quality, food safety and preservation, waste reduction, and protection against chemical and microbiological contaminants [3]. In this context, food can be affected by two main groups of microorganisms: spoilage agents and pathogens [4]. The first group includes bacteria, molds, and yeasts (i.e., *Listeria monocytogenes*, *Escherichia coli*, *Staphylococcus aureus*, *Salmonella* spp., *Bacillus cereus*, *Campylobacter* spp. *Clostridium perfringens*, and *Vibrio cholerae*) capable of modifying the sensory characteristics of food, producing anomalous smells and tastes, changes in color or texture and, ultimately, a reduction in the product’s shelf life [5,6]. Such degradation represents the primary source of losses within the global food supply chain. In fact, the latest available reports estimate that between 25% and 30% (1.3 billion tons/year) of the food produced annually worldwide is wasted due to microbial spoilage [7,8]. In most cases, these spoiled foods only show a reduction in quality, without posing a direct danger to the consumer. However, in some cases, they can lead to food poisoning and epidemic outbreaks, especially in vulnerable populations [9]. Pathogenic microorganisms, on the other hand, present a direct health risk, potentially causing infections, food poisoning, chronic diseases, immunological reactions, or even death at low concentrations [10,11]. These effects can be observed even at low concentrations and after food has been subjected to adverse treatments (i.e., low temperatures, freezing, or partial heat treatments) that inhibit microbial growth [12]. Relevant pathogens include *Salmonella* spp., *Listeria monocytogenes*, *Escherichia coli* O157:H7 y *Campylobacter* spp. [13,14]. Regarding the incidence of these pathogenic microorganisms, in the United States alone, 31 pathogenic species account for an estimated 9.4 million cases of foodborne disease annually [15].

Therefore, the presence of microorganisms in food poses a constant challenge for the food industry. This is why the food industry and the scientific community have made numerous efforts to reduce microbial risk and ensure both food quality and safety. Traditionally, this has been achieved through the large-scale application of synthetic preservatives, including compounds such as butylated hydroxyanisole (BHA), butylated hydroxytoluene (BHT), nitrites, sulfites, benzoates, and sorbates, among others. This practice is characterized by high effectiveness, low production costs, and easy application [16]. When used according to regulatory limits and good manufacturing practices, these additives are generally considered safe. However, some experimental studies under high-exposure conditions have reported transient effects on metabolism, allergic reactions, or alterations in intestinal microbiota [17,18]. Additionally, certain studies suggest that intensive use may contribute to the selection of resistant bacteria in environmental or industrial settings [18,19].

**Figure 1:**
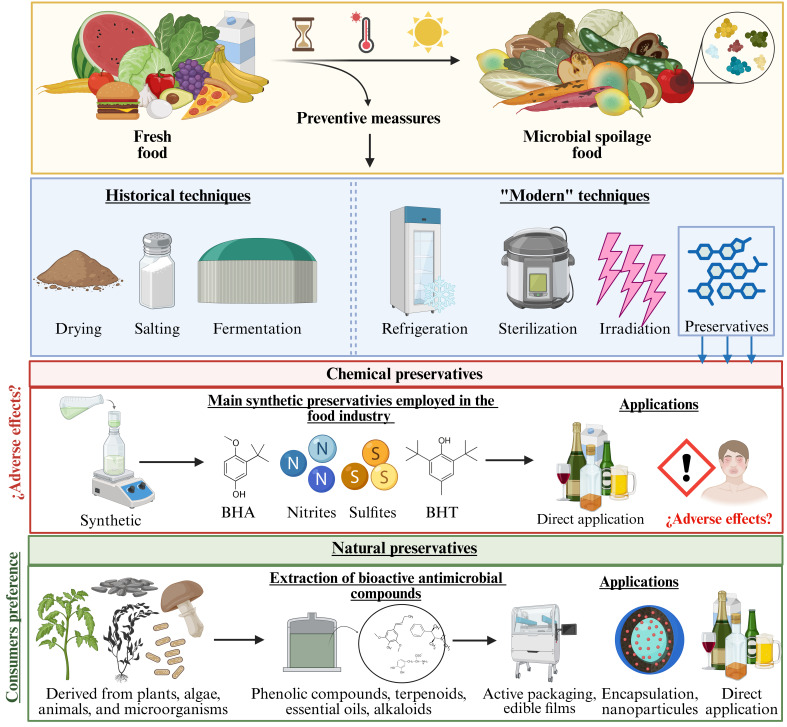
Production of natural preservatives.

Consequently, in recent years there has been an increase in the demand for natural antimicrobial compounds derived from plants, bacteria, fungi, algae, and viruses [20,21], thus recovering historical conservation practices (i.e., traditional use of herbs, spices, and plant extracts) [22]. This antimicrobial activity is attributed to the presence of a diverse profile of secondary metabolites, including significant amounts of phenolic compounds, terpenoids, alkaloids, and essential oils, among others (Figure 1). The magnitude of the antimicrobial effect and its mechanism of action depend on the specific bioactive compound present in the preparation. These mechanisms may include disruption of the cell membrane, interference with nucleic acid synthesis, reduction in the proton motive force, or depletion of ATP, reflecting the diverse ways in which bioactive compounds can inhibit microbial growth [17,23]. Furthermore, some of these molecules can modulate the intestinal resistome, reduce the expression of antibiotic efflux pumps, or interfere with biofilm formation. Therefore, natural agents could offer a safer and more sustainable alternative for biopreservation in food [24,25,26]. In addition, many of these compounds exhibit antioxidant properties, which further contribute to food stability during storage [27].

Therefore, the development of these natural antimicrobials would allow the food industry to adopt a more sustainable model, as it could incorporate circular economy principles [28,29]. This is because many plant-based bioactive compounds can be obtained from agricultural byproducts or food industry waste, transforming biomass that would otherwise be wasted into high-value-added ingredients. In this way, not only is food preservation improved, but waste is also reduced and the use of natural resources is optimized [30]. In this context, this review provides a comprehensive overview of plant-derived antimicrobial compounds, including their principal classes and natural sources, mechanisms of action, and applications in food systems. Beyond summarizing existing knowledge, the manuscript adopts an integrated approach that connects these aspects with current challenges and safety considerations. Particular emphasis is placed on bridging fundamental insights with practical applications, offering an updated and more application-oriented perspective on the use of plant-derived antimicrobials in food preservation.

## 2. Main Classes and Sources of Plant-Based Antimicrobials

**Figure 2:**
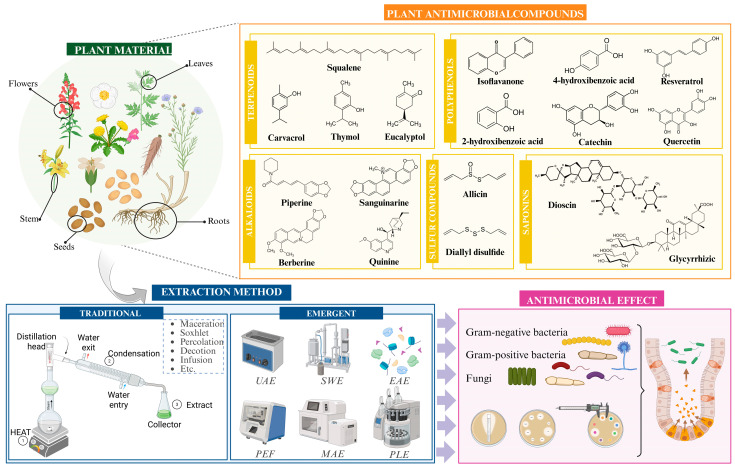
An overview of the main antimicrobial compounds extracted from plant materials. Abbreviations: UAE: ultrasound assisted-extraction; SWE: subcritical water extraction; EAE: enzyme assisted-extraction; PEF: pulsed electric field extraction; MAE: microwave assisted-extraction; PLE: accelerated solvent extraction.

To develop food applications with antimicrobial properties from natural extracts, proper extraction and purification of these compounds is essential beforehand. While this extraction can be carried out from various biological sources, most studies focus on the plant kingdom, as plants represent an accessible and cost-effective matrix with a long history of use. However, despite this background, the safety, regulatory approval, and application of concentrated extracts or isolated compounds still require thorough evaluation before their incorporation into food systems [31,32]. Furthermore, the plant kingdom exhibits remarkable chemical diversity, reflected in the wide range of antimicrobial compounds identified, particularly among secondary metabolites such as essential oils, polyphenols, terpenoids, alkaloids, sulfur-containing compounds, and saponins (Figure 2) [33,34].

EOs are a group of compounds with antimicrobial properties widely documented throughout history. Their traditional use in folk medicine includes, for example, oregano oil (*Origanum vulgare*) as a natural preservative and antibacterial agent, or lavender oil (*Lavandula angustifolia*) used to treat minor wounds and burns [35,36]. Regarding its composition, EOs are complex mixtures of volatile, lipophilic compounds primarily composed of terpenes and phenylpropanoids [37]. In most cases, its antimicrobial properties are attributed to the presence of active monoterpene constituents [38]. Other functional groups reported in plants EOs include alcohols, ketones, aldehydes, and ethers [39]. This chemistry complexity makes complicated to recognize the most potent antibacterial compounds in EOs [40]. As a result, most available studies analyze bioactivity based on the EO-rich extract, without examining the contribution of individual compounds or their specific mechanisms of action. However, it is well established that the antibacterial activity of EOs does not rely on a single mode of action, but rather involves multiple cellular targets that collectively contribute to bacterial inactivation [41]. In this context, investigating the role of individual EO components may provide a deeper understanding of these mechanisms and reveal effects that remain obscured when EOs are studied as complex mixtures. This knowledge could ultimately support the development of more targeted and effective applications in the food industry, as highlighted by numerous studies reporting the antimicrobial properties of EOs derived from a wide range of plant tissues, including stems, peels, bark, leaves, fruits, and seeds. Some examples can be seen in Table 1. However, these results reported in Table 1 are obtained using different antimicrobial assay methods (e.g., MIC, inhibition zone, CPSI), which are not directly comparable. MIC values provide quantitative measurements of antimicrobial potency, whereas inhibition zones depend on diffusion conditions and should be interpreted qualitatively. Therefore, comparisons should be made cautiously and preferably within the same assay type. Regardless of the method used, many of these results correspond to extracts that are already commercially available.

Of all the compounds present in EOs, one of the families that has been the focus of the most studies are terpenoids. This class of compounds is characterized by having a large hydrocarbon structure composed of 5-carbon isoprene units [80]. These compounds have shown effectiveness against diseases caused by drug-resistant bacteria. However, to observe these effects, several problems must be overcome. The first is that this class of compounds has demonstrated limited effectiveness against some types of bacteria. The second limitation is that, in many cases, the concentrations of terpenoids needed for significant activity are high (millimolar). One possible solution may be the combination of several terpenoids [81,82]. For example, the combination of carvacrol and thymol has bacteriostatic and bactericidal activities when used at low concentrations for 5–10 min [81]. The combination of squalene (leaves of *Syzygium samarangense*) and globulol (leaves of *Syzygium malaccense*) was associated with strong antimicrobial activity, including inhibition of *E. coli* growth (MIC = 3.75 µL/mL) and a reduction in *Candida albicans* biofilm formation by over 80% [54]. In another study, the combination of terpenoids such as artemisia ketone (43.19%) with different terpenes (caryophyllene (15.75%), *β*-selinene (10.32%), and germacrene D (9.56%)) demonstrated strong antibacterial activity against *E. coli* (inhibition zone of 75.67 mm; MIC 5.34 µg/mL) and notable antifungal effects [61]. Other terpenoids of interest include eucalyptol, *α*-terpineol, *α*-eudesmol, (−)-bornyl-acetate, and linalool, among others (Table 1). Compared with other phytochemical classes, EOs (including terpenoids) typically provide the most potent inhibition at low concentrations. However, their inherent volatility, hydrophobicity, and strong aroma present major practical challenges for direct inclusion in food matrices; these factors frequently necessitate encapsulation or delivery vehicles to improve solubility, stability, controlled release, sensory acceptability, and shelf-life [83,84]. Moreover, although many EOs components are regarded as Generally Recognized as Safe (GRAS), individual constituents (e.g., pulegone, thujone) have demonstrated toxicity at high doses, and safety assessments specific to their use as food preservatives remain incomplete [85]. It is also increasingly recognized that subinhibitory concentrations can induce adaptive responses in microorganisms, potentially contributing to tolerance and complicating long-term efficacy [83]. These constraints (sensory impact, regulatory ambiguity, dose-dependent toxicity and variable performance in complex food systems) underscore the need for integrated formulation strategies and rigorous in situ testing prior to industrial applications. In addition to terpenoids, plants produce other secondary metabolites of significant antimicrobial interest, most notably phenolic compounds. This group has been the subject of numerous studies in recent decades due to its structural diversity and functional relevance. Phenolic compounds are broadly classified according to their structure into flavonoids and non-flavonoids [86,87,88]. Flavonoids are characterized by a carbon backbone comprising two phenyl rings and a central heterocyclic ring [87]. This subclass can be further divided into flavones, flavonols, flavanones, and isoflavonoids, depending on the degree of hydrogenation and substitution of the heterocyclic ring. In contrast, non-flavonoid polyphenols include phenolic acids, lignans, stilbenes, and other structurally diverse compounds [86,87]. The primary sources of antimicrobial polyphenols for industrial applications include peels, seeds, leaves, and husks of diverse plants (Table 1). Some of these applications are not recent, as for example, various extracts with antimicrobial activity (e.g., *Salvadora persica* L., or *Rosmarinus officinalis*) have been traditionally used in folk medicine as resources for oral hygiene [86,89,90,91]. Furthermore, it has been observed that the efficacy of polyphenolic extracts against various microorganisms is conditioned by their lipophilic nature, as this promotes molecular interactions with the microorganism’s cell membranes [92]. In general (Table 1), a moderate increase in lipophilicity improves antimicrobial activity by facilitating membrane insertion, although excessive levels can reduce it by decreasing the compound’s solubility in aqueous media. Moreover, their efficacy is often markedly reduced in situ due to strong interactions with food constituents (proteins, fats, carbohydrates) that sequester bioactive molecules and limit availability at the microbial interface, and their antimicrobial activity in actual food matrices is frequently much lower than in simplified in vitro systems [93]. Moreover, phenolics exhibit variable bioavailability, are susceptible to degradation (oxidation, heat, pH) during processing/storage, and can impart undesirable organoleptic changes (bitterness, astringency) at concentrations required for antimicrobial efficacy [94]. Regulatory approval for phenolic use as direct preservatives is currently limited, with only select compounds authorized in specific authorities, and toxicological evaluations are incomplete for many candidates [93]. Consequently, while plant phenolics hold significant potential, feasibility of application in diverse food systems remains constrained, necessitating targeted delivery strategies (e.g., encapsulation, active packaging) and comprehensive safety and performance profiling.

Another important group of secondary metabolites present in plants are alkaloids, nitrogenous compounds with heterocyclic chemical structures characterized by their high biological activity [95]. According to their structure, alkaloids can be classified in isoquinoline, quinoline, indole, piperidine, pyrrolidine, tropane, and purine alkaloids [96]. They can be recovered from different natural sources, including the roots, leaves, barks, and seeds of different plants [95]. Numerous studies have shown that alkaloids possess a wide range of antimicrobial properties, acting against Gram-positive and Gram-negative bacteria, as well as pathogenic fungi. Among the antimicrobial alkaloids that have been most extensively studied, berberine, quinine, sanguinarine, and piperine are particularly noteworthy [97]. For example (Table 1), berberine, extracted from the root of *Berberis vulgaris*, displays significant activity against *S. aureus* and *E. coli* [98]. Similarly, quinine, obtained from the bark of *Cinchona officinalis*, has demonstrated antifungal effects against *Candida albicans* [63]. Therefore, alkaloids have a wide antibacterial spectrum with a good antibacterial effect on common clinical strains, including drug-resistant bacteria [96]. However, the bioavailability of alkaloids is a common pharmacokinetic concern. Many alkaloids can form water-soluble salts with acids, while their free base forms are lipophilic, facilitating absorption via passive diffusion across the gastrointestinal epithelium [99]. In fact, several alkaloids exhibit significant toxicity toward mammalian cells, and certain subclasses (e.g., pyrrolizidine alkaloids) are recognized for their hepatotoxic and genotoxic effects, raising safety concerns for food preservation uses [100]. However, the bioavailability of alkaloids in complex food matrices is often unpredictable, with interactions with proteins and fats reducing effective concentrations at microbial targets and complicating dose standardization across food types [101]. Therefore, careful dosage control and safety assessment of alkaloids are critical, and they may face stricter regulatory scrutiny compared with polyphenols or EOs. In fact, regulatory frameworks in many authorities do not support the inclusion of alkaloids as direct food preservatives, meaning that despite their potent biochemical activity, practical feasibility in diverse food systems is constrained by safety, sensory and regulatory limitations. Another group of secondary metabolites of interest are sulfur-containing compounds, commonly present as thiols, sulfides, disulfides, or sulfones. These metabolites are predominantly found in plants of the genus *Allium*, including garlic and onion, and are typically extracted from bulbs, roots, or damaged tissues. Numerous studies have demonstrated that these compounds exhibit notable antimicrobial activity against bacteria, fungi, and protozoa [102]. For example (Table 1), allicin, derived from garlic (*Allium sativum*), inhibits the growth of *S. aureus*, *S. enterica* and *C. albicans* [67,103]. Likewise, diallyl disulfide has shown bactericidal effects against antibiotic-resistant strains [104]. However, their chemical reactivity can compromise stability during storage or processing. Moreover, although effective, their practical use may be limited by sensory and regulatory constraints, making tailored formulation strategies essential [105,106]. Moreover, the toxicological profile of some sulfur compounds remains under characterized when consumed at levels necessary for antimicrobial function, and regulatory acceptance as food preservatives is limited [107]. These constraints underscore that while sulfur-containing compounds have mechanistic appeal, their stability, sensory impact and regulatory feasibility must be carefully balanced against antimicrobial benefits in practical food systems.

Saponins represent another group of secondary metabolites with significant biological relevance (Table 1). Structurally, these molecules are glycosides characterized by hydrophilic sugar (glycone) units attached to hydrophobic aglycone cores [108,109]. They can be extracted from roots, seeds, and leaves of various plant species and have demonstrated antimicrobial activity by altering the permeability of cell membranes in pathogenic bacteria and fungi [108,109]. In addition, saponins have been shown to enhance the efficacy of conventional antibiotics, either by increasing microbial membrane permeability or by interfering with biofilm formation [110]. However, in many cases these compounds must be encapsulated so that their consumption does not damage the intestinal mucosa or disrupt blood cells [111]. Encapsulation can also enhance consumer acceptance, as these compounds are often associated with a pronounced bitter taste [112], and can help preserve their antimicrobial efficacy, which might otherwise be reduced due to interactions with food components arising from their amphiphilic nature [109].

In conclusion, plant extracts encompass a wide array of secondary metabolites exhibiting antimicrobial activity against diverse pathogenic microorganisms. Their effective application depends on a comprehensive understanding of the mechanisms underlying their bioactivity, coupled with careful evaluation of their safety and efficacy across different microbial targets, as well as consideration of factors such as extraction, concentration, stability, and interaction with the surrounding matrix.

## 3. Modes of Antimicrobial Action of Plant-Derived Compounds

**Figure 3:**
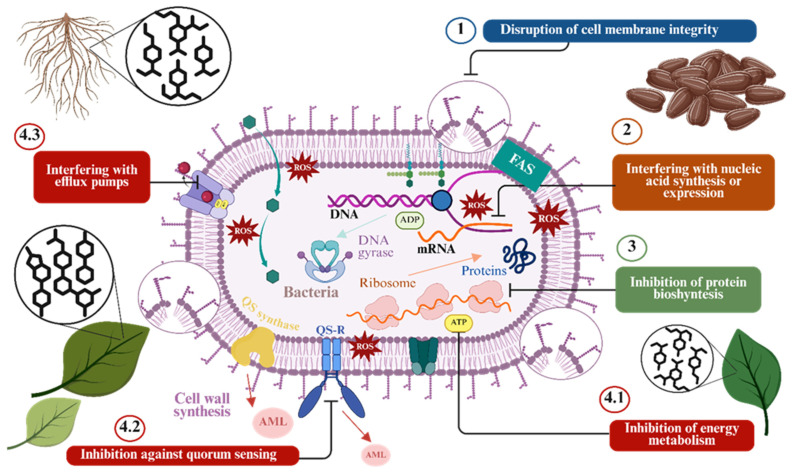
Major antibacterial mechanism of action of plant phytochemicals at the cellular level. Abbreviations: ROS: Reactive oxygen species; FAS: Fatty acid synthase (biosynthesis pathway); AML: Antimicrobial lipid.

A large body of research has examined the antimicrobial properties of plant-derived phytochemicals [78]. However, their exact modes of action (MOA) remain only partially understood [113,114]. Phytochemicals can act on microbial cells through multiple pathways, producing additive, synergistic, or antagonistic effects by interacting with one or several cellular targets [115]. Currently, the best-known MOA of antimicrobial plant compounds are related to disruption of cell walls, inhibition of DNA and protein synthesis, and disruption of metabolic pathways, among others (Figure 3).

### 3.1. Cell Wall Disruption

Among the various mechanisms through which phytochemicals exert antimicrobial effects, disruption of microbial cells stands out as particularly significant, as evidenced by numerous studies. Extensive experimental data (Table 1) demonstrate that plant-derived compounds (especially EOs and amphiphilic phenolics) interact with lipid bilayers, increase membrane permeability, induce leakage of intracellular contents, and ultimately cause cell lysis [116]. These effects have been observed in both Gram-positive and Gram-negative bacteria using microscopy, dye leakage assays, and ion efflux measurements. For example, phenolic compounds such as bisdemethoxycurcumin exert membrane-disruptive effects via multiple converging pathways, including direct interaction with phospholipid bilayers, lipid peroxidation triggered by ROS, and alterations in membrane potential or permeability [117]. 3-*p*-trans-coumaroyl-2-hydroxyquinic acid, a phenolic compound isolated from needles of *Cedrus deodara*, induced a significant increase in the proportion of cells in the fluorescence region, suggesting an increase in the number of membrane-damaged bacterial cells together with substantial hyperpolarization, disruption of membrane integrity, profound morphological alterations, and engagement with membrane-associated proteins and lipids [118].

In terpenoids, the phenolic-OH moiety is essential for this effect as neither the *O*-methyl derivatives nor the benzylic derivates were as effective. Therefore, perturbation of ion homeostasis upon increase in cell wall permeability is key for the action of terpenoids against bacteria cells [119]. Some terpenoids with demonstrated efficacy include carvacrol and thymol, which rapidly disrupt the membrane integrity of both Gram-negative and Gram-positive bacteria, causing ion leakage [119,120]. Other terpenoids such as linalool can induce pore formation or alter membrane fluidity by disrupting membrane integrity through interactions with membrane proteins and phospholipids, significantly affecting cellular homeostasis. For example, *Brochothrix thermosphacta* was significantly inhibited by linalool (MIC 1.5 mL/L). The antimicrobial action of linalool was primarily attributed to its disruptive effects on the bacterial cell wall structure, leading to compromised integrity and the subsequent efflux of intracellular components such as alkaline phosphatase, proteins, nucleic acids, and ions. This disruption of the cell wall barrier not only facilitated cellular leakage, but also contributed to downstream effects on cellular metabolism, as reflected by alterations in key enzymatic activities, including pyruvate kinase, malate dehydrogenase, and ATPase [121].

Similar effects have been detected among different terpenes isolated in different essential oils. For example, treatments with limonene, *β*-citronellol, carvone, and carvacrol cause extravasation of cellular materials in *E. coli* and *S. aureus* [122]. In fact, combined terpene treatments modified the bacterial outer membrane and overall cellular architecture, increasing membrane permeability. This disruption of osmotic balance led to enhanced leakage of intracellular components. Consequently, the observed loss of regular morphology, structural cohesion, and osmotic regulation following combined terpene exposure suggests that the bacterial cell wall and outer membrane are likely primary targets of these compounds [123].

Another example includes several organic acids (e.g., L-malic acid, succinic acid) and flavonoids (e.g., isoquercitrin) isolated from *Pingyin rose* bugs that can cause irreversible damage to the membrane structure of *S. aureus* at concentrations as low as 0.39 mg/mL [76]. Notably, the antimicrobial activity of organic acids is primarily attributed to their undissociated form, which can more readily penetrate the cell membrane and disrupt internal homeostasis [124]. In another study, membrane disruption was attributed to the permeabilization of microbial cell membranes by several bioactive polypeptides (thionins NsW1 and NsW2) isolated from the medicinal plant *Nigella sativa*. These peptides lead to bacterial and fungal lysis, highlighting multiple mechanisms through which antimicrobial compounds can act. These compounds exhibit potent antimicrobial effects, particularly against Gram-positive bacteria such as *S. aureus* and *Bacillus subtilus* (MIC 6.5 and 3.25 µM, respectively) and the fungus *C. albicans* (MIC 3.25 µM). In contrast, *N. sativa* extracts were ineffective against the Gram-negative bacterium *E. coli*, presumably due to the protective effect of its lipopolysaccharide-rich outer membrane, highlighting the influence of cell wall composition on susceptibility [75]. These mechanisms of action were verified by tests of potassium ion efflux, salt tolerance, extravasation of cellular contents, absorption of crystal violet, pH drop (pH_int_), and morphological changes analyzed by electron microscopy [122,125,126].

### 3.2. Inhibition of DNA Synthesis

In other cases, phytochemicals act by targeting microbial nucleic acid synthesis as supported by direct biochemical evidence for certain alkaloids, polyphenols, and sulfur-containing compounds, which can intercalate into DNA or inhibit topoisomerases and DNA polymerases. For example, polyphenols such as epigallocatechin gallate and theaflavin-3,3′-digallate cause DNA segregation and cell division on *C. perfringens* [127]. Other polyphenols (e.g., flavonoids) are also capable of inhibiting DNA replication and the activity of topoisomerase IV, an enzyme involved in supercoiling and DNA segregation [128]. Moreover, some flavonoids can inhibit the DNA gyrase against Gram-negative bacteria, demonstrating measurable antibacterial activity, whereas their effects on Gram-positive bacteria are primarily associated with membrane-targeted mechanisms and exhibit comparatively lower antibacterial potency [129].

For other compounds, such as some terpenoids, this mechanism is proposed based on indirect observations, including cell cycle arrest or reduced replication rates, and requires further validation to confirm a direct effect on DNA metabolism. For example, terpenoids (e.g., thymol and carvacrol) can downregulate ribosomal proteins and decreased incorporation of labeled precursors into RNA and proteins [130]. Regarding terpenes, their antimicrobial activity is primarily associated with the disruption of replication and transcription processes. For example, andrographolide exhibits inhibitory activity against microbial DNA synthesis, reducing thymine incorporation by more than 65%. It also suppresses RNA synthesis by approximately 75% and protein synthesis by 64%, while exerting no significant effect on cell wall biosynthesis [57]. Similarly, oridonin interferes with protein and DNA metabolism and induces alterations in bacterial morphology, collectively impairing bacterial viability [59]. Terpinen-4-ol might also block protein synthesis by inhibiting DNA synthesis as fluorescence intensity of treated groups was weaker than that of the control group and some protein bands of bacteria treated with terpinen-4-ol became fainter. However, targeting DNA and proteins can exert selective pressure on bacteria, whether alive or dead, potentially promoting the development of drug resistance like conventional antibiotics. Therefore, the clinical use of phytochemicals should be carefully controlled [56]. Other examples include alkaloids such as berberine which can intercalate into DNA molecules, thereby preventing replication and transcription. Moreover, berberine-DNA complexes have been reported to induce oxidative damage to guanine bases upon photo-irradiation, leading to replication stress and double-strand breaks in susceptible organisms [128,131].

### 3.3. Inhibition of Protein Synthesis

Inhibition of protein synthesis represents another crucial antimicrobial strategy of different phytochemicals, which impair translation by acting on different components of the ribosomal machinery [132]. Natural compounds such as terpenes, terpenoids, alkaloids, and organosulfur molecules have been shown to disrupt translation through diverse mechanisms. However, it is also possible to find some studies on the potential impact of phenolic compounds on protein synthesis. This is the case of epigallocatechin gallate and lavandulylated flavonoids. The first compound affected the proteins involved in the septum formation and inhibits the Z-ring formation in *C. perfringens* [127], whereas lavandulylated flavonoids altered the large-conductance mechanosensitive channel (MscL) and YidC2 proteins by inhibiting MscL function and targeting the membrane protein translocase YidC2. Consequently, MRSA undergoes alterations in osmosis pressure and disruption of cell membrane integrity [49]. This disruption leads to incomplete or dysfunctional proteins, ultimately compromising cellular functions and viability.

Regarding terpenes, andrographolide and oridonin demonstrated an inhibitory capacity on protein synthesis of 36% and 27.51% respectively in *S. aureus*, resulting in the downstream biosynthetic pathway inhibition [57,59]. Ursolic acid was also able to reduce protein synthesis in *S. aureus* by interacting with proteins involved in translation fidelity, ribonuclease and chaperone subunits, as well as proteins associated with glycolysis and oxidative stress responses [133]. In the case of *Streptococcus agalactiae*, protein synthesis was generally inhibited in a time depend manner by terpinene-4-ol by inhibiting DNA synthesis [56]. Moreover, some terpenoids present in essential oils also contribute to this inhibitory effect. Proteomic analyses of bacterial cells treated with, for example, lemon essential oil, reveal a marked reduction in abundance of proteins related to translation [134,135]. Therefore, although terpenoids are often credited with disrupting cell membranes, these results demonstrate their capacity to suppress the synthesis of ribosomal and other translation-associated proteins.

Likewise, several plant-derived alkaloids (e.g., chelerythrine, berberine) interfere with the translational apparatus. Chelerythrine disrupts bacterial protein expression and synthesis, inhibiting protein biosynthesis and causing leakage of proteins from the cell, which ultimately leads to bacterial cell death [66]. Concerning berberine, the evidence suggests that this molecule associates with the 50S ribosomal subunit, impairing peptide bond formation, while other alkaloids may hinder tRNA binding or inhibit the activity of aminoacyl-tRNA synthetases [115,136]. These interactions block key steps in translation.

Regarding sulfur compounds, allicin stands out, a molecule for which there are different studies that support its ability to inhibit protein synthesis. Allicin readily reacts with cysteine residues in proteins, provoking thiol stress and disturbing the cellular redox balance. Consequently, translation rates are significantly reduced; for instance, exposure of *E. coli* to allicin causes a substantial drop in the amount of newly synthesized proteins [137]. In addition, allicin exerts a partial inhibitory effect on RNA polymerase, thereby coupling transcriptional inhibition with diminished protein synthesis [138,139].

### 3.4. Disruption of Metabolic Pathways

Phytochemicals can exert antimicrobial effects not only through membrane damage, nucleic acid interference, or inhibition of protein synthesis, but also by perturbing essential metabolic pathways in bacteria and fungi. This mechanism targets core biochemical processes such as energy production, central carbon metabolism, and enzymatic functions, ultimately leading to metabolic collapse. While effects such as ATP depletion and altered enzymatic activities are well documented for essential oils and specific polyphenols, other proposed metabolic disruptions are indirectly inferred from global metabolomic changes or growth inhibition under defined conditions, highlighting areas for further experimental study.

Concerning phenolic compounds, they can bind to bacterial enzymes that require metal cofactors (such as iron or zinc), thereby inhibiting their activity and destabilizing metabolic pathways [140]. For instance, quercetin and kaempferol have been reported to inhibit ATP synthase in *E. coli*, thus impairing the production of cellular energy [141]. Other polyphenols (e.g., eugenol, cinnamic acid) can inhibit the activity of key enzymes involved in the biosynthesis of peptidoglycan, an essential component of bacterial cell walls [142]. This inhibition leads to weakened structural integrity and increased susceptibility to osmotic pressure. Additional inhibited enzymes include those involved in the tricarboxylic acid (TCA) cycle, glycolysis, and fatty acid biosynthesis. Disruption of these pathways impairs energy production and macromolecular synthesis, thereby restricting microbial growth [143]. Other reported mechanisms reported among phenolic compounds include aerobic metabolism interference (i.e., caffeic acid in *S. aureus*) [144], or reduction in biosynthetic and metabolic activities leading to induction of the bacterial cell death system (i.e., theaflavin-3,3′-digallate in *C. perfringens*) [127]. Additionally, flavonoids have been shown to inhibit bacterial efflux pumps, which are responsible for expelling toxic substances and antibiotics out of the cell. This inhibition increases intracellular drug concentrations and enhances the efficacy of antimicrobial treatments [145].

Furthermore, essential oil components often interfere with ATP homeostasis. For instance, several phytochemicals inhibit the activity of ATP-dependent enzymes (e.g., ATPase), leading to a drop in ATP levels [114]. This decline in ATP undermines the bacteria’s ability to power vital metabolic processes, contributing to their death. In some cases, the lipophilic phytochemicals directly bind to ATP synthase, blocking its function [146]. One well-documented example comes from studies on garlic essential oil, which inhibits the activity of key enzymes in the TCA cycle, such as succinate dehydrogenase, and suppresses pyruvate kinase, which links glycolysis to the TCA cycle in *Pseudomonas fragi*. These disruptions reduce cellular respiration and energy generation, as shown by metabolomic profiling that revealed alterations in respiratory chain metabolites, amino acid biosynthesis, and nucleic acid metabolism after garlic essential oil exposure [147].

Beyond energy metabolism, some plant-derived compounds can inhibit quorum sensing (QS) regulated processes, such as biofilm formation, by downregulating the expression of genes involved in extracellular polysaccharide (EPS) biosynthesis and transcriptional regulators [148], as observed for coumarins from *Nicotiana* spp. in *Ralstonia solanacearum* [79]. This gene-level interference reduces EPS production, a key factor in biofilm development and bacterial virulence. In addition to direct QS modulation, bacterial resistance can limit the efficacy of phytochemicals through overexpression of efflux pumps that actively remove antimicrobial agents from the cell [149]. These pumps belong to diverse families, including the resistance-nodulation-division (RND) family, ATP-binding cassette (ABC) transporters, and the major facilitator superfamily (MFS). By lowering the intracellular concentration of active compounds, efflux pumps diminish their antimicrobial effectiveness. Moreover, some bacteria employ these mechanisms in combination with others, such as target site modifications or enhanced biofilm production, further enhancing their survival against phytochemical exposure [150]. For example, resveratrol from Pinot Noir grape extract exhibits antimicrobial activity against *Campylobacter jejuni*, which is potentiated by the efflux pump inhibitor phenylalanine-arginine β-naphthylamide (PAβN). This observation highlights that the extract’s efficacy is limited by efflux-mediated extrusion rather than by direct disruption of QS [151]. Collectively, these findings illustrate that the interaction between phytochemicals and bacterial adaptive strategies involves multiple layers of regulation, including both QS modulation and active compound efflux.

In summary, phytochemicals exert antimicrobial activity through a diverse array of mechanisms that collectively compromise microbial viability. These multi-target effects act synergistically, simultaneously compromising multiple cellular processes, which enhances overall antimicrobial efficacy and limits the ability of microorganisms to develop stable resistance [152]. At the same time, subinhibitory exposures can induce transient stress responses, such as upregulation of efflux pumps or other adaptive pathways, particularly under prolonged or repeated use. This highlights that, despite their multi-target nature, careful attention to formulation, dosing, and delivery strategies is required to maintain effective concentrations and prevent tolerance development [153]. By understanding the molecular targets and interactions of phytochemicals, it will be possible not only to rationally design novel antimicrobial formulations but also to strategically combine compounds to maximize synergistic effects while minimizing the risk of resistance. These features underscore the promise of plant-derived compounds as versatile alternatives or complements to conventional antimicrobials in both food preservation and therapeutic contexts.

## 4. Antimicrobial Compounds Applications in the Food Industry

### 4.1. Synthetic Preservatives in the Food Industry

There are several chemical preservatives routinely used in the food industry. The main classes are nitrates, organic acids and their salts, sulfites, and specific antifungal agents (Table 2). Their consumption is associated with health risks such as hypersensitivity, allergies, asthma, hyperactivity, neurological damage, and cancer [154]. For example, nitrates and nitrites, widely used in the meat industry to inhibit the growth of pathogenic organisms and improve the sensory properties of products, substantially increase the risk of cancer, methemoglobinemia, gastroenteritis, thyroid gland enlargement, and diabetes mellitus. In fact, excessive consumption of these additives is currently considered one of the biggest public health problems [155]. However, according to the latest scientific evidence, its responsible use not only does not represent a health risk, but could even exert some beneficial effects on the body, including protection of the cardiovascular system, control of blood pressure, and homeostasis of the arteries [156].

Another group of antimicrobial food additives that has been the subject of much research in the last decade includes certain organic acids and their salts. For example, high consumption of benzoates (benzoic acid and salts such as sodium benzoate), used primarily in processed food and soft drink production, is associated in children with an increased risk of asthma, allergies, or attention deficit hyperactivity disorder [159]. Additionally, high benzoate consumption may cause glycine shortage, which negatively impacts brain neurochemistry [155]. In adults, its high consumption is associated with mutagenic effects, generate oxidative stress, disrupt hormones, and reduce fertility [159]. In contrast, sorbic acid and its corresponding salts (mainly potassium sorbate), used mainly in bakery and pastry products, dairy products, beverages, sauces, meat products, preserves and processed vegetable foods, can generate oxidative stress, cell damage, irritation, and allergies at elevated levels [160]. Furthermore, several studies indicate that ingesting considerable amounts of potassium sorbate (>25 mg/kg) can generate mutagenic substances capable of causing DNA damage, chromosomal alterations, sister chromatid exchange, and other genotoxic and cytotoxic effects. These alterations, in turn, have been linked to an increased risk of diabetes, cancer, and other chronic diseases [161]. Regarding propionates, these additives are particularly effective against mold growth and are widely used in industrial baking to prevent rancidity and mold in doughs and breads. Regulations and scientific reviews generally classify them as safe when used according to good manufacturing practices, although some literature investigates acute effects on human metabolism [162]. Some experimental studies under high-exposure conditions have reported transient effects on glucose metabolism or hormonal responses; however, such effects have not been observed at typical consumption levels [163]. Sulfites and sulfating agents, including sulfur dioxide, sodium and potassium sulfite, metabisulfite, and bisulfites, are used in canned goods and in wine production to inhibit malolactic fermentation [164]. While certain individuals may experience sensitivity reactions, such as headaches or bronchoconstriction in asthmatic patients, these effects are generally associated with higher exposures than those occurring under standard food-use conditions [107,165,166]. Similarly, antifungal agents such as natamycin (or pimaricin) are applied to the surface of cheeses and some sausages to prevent mold and yeast growth. Due to its low solubility, natamycin remains primarily on the surface, and studies indicate a favorable safety profile when used in accordance with authorized food-use regulations [167]. Collectively, these findings highlight that, while potential risks have been identified under experimental or high-exposure conditions, synthetic preservatives remain effective and safe when used as intended in food systems.

### 4.2. Applications of Natural Antimicrobials Compounds in Food Industry

The increasing demand for healthier, more natural, minimally processed foods has driven interest in the use of various plant-based antimicrobial compounds as alternatives to synthetic preservatives. These compounds, including essential oil constituents, polyphenols, terpenoids, and organic acids, have shown significant activity against a range of pathogenic and spoilage microorganisms through distinct mechanisms of action, making them promising candidates for food preservation [168]. Additionally, many of these natural compounds exhibit complementary functional properties, such as antioxidant capacity, which can help limit deterioration processes like lipid oxidation in meat or browning in fruit [169,170]. Advances in formulation science and food technology are contributing to improved efficacy, stability, and applicability of these natural preservative systems, suggesting that their use in commercial foods may increase in the future. This approach offers a potential strategy to support food safety, quality, and sustainability, while acknowledging the challenges that remain in their practical implementation.

#### 4.2.1. Incorporation into Meat Products

Meat and poultry products are highly perishable due to microbial growth and oxidative spoilage. Traditional cures (e.g., nitrite in cured meats) effectively control pathogens like *Clostridium botulinum* but have been associated with potential health risks, motivating the scientific investigation of natural substitutes with comparable efficacy [171]. Plant-derived compounds such as essential oils from herbs and spices (Table 3) are widely studied as natural antimicrobials and antioxidants in meat products [171]. These bioactive properties are attributed to the presence of various bioactive compounds within the extracts, particularly phenolic constituents such as thymol and carvacrol. These phenolic compounds have been shown to disrupt bacterial cell membranes, thereby exerting inhibitory effects against foodborne pathogens such as *L. monocytogenes*, *Salmonella* spp., and various spoilage bacteria [171]. An illustrative example of this is the application of thyme essential oil, which is particularly rich in thymol. When incorporated into beef patties at a concentration of 1% (*w*/*v*), it effectively reduced *E. coli* counts from 23 MPN/g to undetectable levels over 14 days of storage at 4 °C, exhibiting antimicrobial efficacy comparable to that of conventional preservatives such as synthetic nitrite [171]. In another study, chestnut inner shell extracts rich in phenolic compounds and flavonoids applied at 2 mg/mL were incorporated into chicken meat, and an inhibition of *C. jejuni* growth was observed [171,172]. Organic acid-based marinades and herbal extracts have demonstrated significant efficacy in reducing surface contamination on poultry products. For example, the combination of thyme essential oil with an acidic marinade composed of lemon juice and *Yucca schidigera* extract markedly enhanced the inactivation of *S. enterica* on raw chicken breast during the marination process [173]. Similarly, essential oils derived from rosemary and basil have been shown to suppress both *Salmonella* spp. and spoilage-associated bacteria when applied as surface coatings or immersion dips on poultry meat [174]. These natural treatments can lead to immediate bacterial reductions of 2–4 log CFU/g on poultry surfaces and slower microbial growth during storage [174]. This inhibitory capacity will depend on the composition of the matrix, particularly its high protein and lipid content. Interactions with proteins and fats can reduce the availability of active compounds by binding or partitioning into the lipid phase, thereby decreasing antimicrobial efficacy compared to in vitro conditions [175]. In addition, oxidative processes and microbial growth occur simultaneously in meat, requiring compounds with both antimicrobial and antioxidant activity. To address these limitations, technological approaches such as nanoemulsions and encapsulation have been widely explored, as they improve dispersion, protect active compounds, and enable controlled release [176]. These systems have demonstrated the ability to extend shelf life and reduce microbial load in meat products under realistic storage conditions. Hence, the integration of natural antimicrobials into meat products offers an effective strategy to enhance microbial safety without adversely affecting flavor when used at optimal concentrations [171]. Their multifunctional properties support both pathogen inhibition and quality preservation. Future studies should focus on optimizing application methods and exploring synergistic effects with existing preservation techniques. This approach presents a promising avenue for sustainable and consumer-friendly meat product preservation.

#### 4.2.2. Incorporation into Horticultural Products

Postharvest deterioration of horticultural products is primarily caused by fungal decay (e.g., *Botrytis* spp., *Penicillium* spp.), bacterial spoilage, moisture loss, and enzymatic browning. To mitigate these spoilage mechanisms and preserve quality, various preservation strategies have been developed, among which incorporation of bioactive compounds into horticulturally derived products stands out as an effective method (Table 3). For example, the addition of pomegranate, rosemary, lemon, and balsamic lemon essential oils to jam had proved to be an effective way of reducing counts of mesophilic aerobes, yeasts, molds, and coliforms compared to the control jam. In fact, the minimum inhibitory concentration was reported as low as 0.0005 g/mL for complete inhibition of visible microbial growth [233]. Similarly, pomegranate extract, vanillin, and geraniol were added to strawberry juice, demonstrating that, at the concentrations tested, these treatments effectively enhanced microbial safety while maintaining juice quality [234]. These studies demonstrate that natural preservative strategies, especially incorporation of antimicrobials (such as essential oils, organic acids, and antimicrobial peptides) can effectively reduce decay and prolong the postharvest life of fruits and vegetables derived products while often improving or maintaining sensory properties.

However, the efficacy of these compounds is governed by multiple factors, including extract type and concentration, interactions with the food matrix, and environmental parameters such as surface characteristics, water activity, and the presence of native microflora [235]. Unlike in vitro systems, antimicrobial agents may be unevenly distributed on food surfaces or rapidly diluted, reducing their effectiveness. Moreover, interactions with plant tissues and natural enzymes can lead to degradation or reduced activity. To overcome these challenges, coating technologies and nanoemulsion-based delivery systems have been developed, allowing better adhesion to surface and sustained antimicrobial action. For instance, nanoemulsion coatings have shown the ability to significantly reduce microbial populations on fresh produce while maintaining quality attributes [175]. Accordingly, each application must be carefully optimized (dose, stability, sensory impact) to ensure both microbial safety and acceptable product quality.

#### 4.2.3. Incorporation into Bakery Products

Baked goods with elevated water activity, such as bread and cakes, are particularly susceptible to fungal spoilage, which significantly limits their shelf life. Under ambient storage conditions, molds from the genera *Aspergillus* and *Penicillium* can rapidly develop on these products, leading to visible degradation, off-odors, and compromised sensory quality. In these high-moisture systems, the effectiveness of plant-derived antimicrobials is often reduced compared to in vitro conditions due to interactions with the food matrix, including binding to proteins and starch, which can limit the availability of active compounds [236,237]. In contrast, bakery products with lower water activity, such as biscuits and crackers, are less prone to microbial growth but may also restrict the diffusion and activity of antimicrobial agents. As a result, effective preservation strategies are essential to maintain product stability and ensure consumer safety [236]. Traditionally, bakers use chemical preservatives (e.g., calcium propionate, sorbic acid) to inhibit mold [236]. Given growing consumer concerns over synthetic additives and their potential health effects, there is increasing interest in the use of natural alternatives for bakery products preservation. Natural preservation strategies for baked goods focus on antifungal agents and techniques to replace these additives. In recent years, researchers have explored plant-derived antifungals, notably essential oils, and spice extracts, to keep baked products mold-free (Table 3).

It has been shown that many essential oils have strong antifungal efficacy in bread without toxic effects [219]. Among them, clove and cinnamon oils have shown strong antifungal activity against common bread molds. Notably, the incorporation of clove and orange essential oils into bread formulations allowed loaves to be stored at 20 °C for up to 7 days without visible mold growth or quality loss, whereas untreated control samples developed mold in approximately 4 days [217]. More examples can be found in Table 3. Hence, natural antimicrobials offer a targeted solution to the predominant challenge of fungal spoilage in bakery products, especially those with high water activity. Unlike other food matrices, baked goods require preservation strategies that withstand thermal processing and maintain functionality post-baking. The development of heat-stable, food-grade delivery systems is therefore critical. Future research should focus on integrating these compounds without compromising dough rheology, texture, or flavor profiles.

#### 4.2.4. Development of Food Packaging Materials and Edible Coatings

Another widely studied strategy for applying natural antimicrobials involves using edible coatings and active packaging as carriers for these compounds. Edible coatings consist of thin layers of edible materials such as polysaccharides (e.g., chitosan or alginate), proteins (such as casein or soy protein), or lipids and waxes, which are applied directly to the surface of food products [238]. By forming a semi-permeable barrier, these coatings not only enable the controlled release of antimicrobial agents but also help reduce moisture loss and limit oxygen exposure, contributing to the preservation of food quality and stability [239]. This approach often extends shelf life and preserves quality without significantly affecting the product’s interior [168].

Their effectiveness in extending shelf life and limiting microbial spoilage has been well documented in numerous studies (Table 3). For example, a chitosan-based film on fresh fish or meat can gradually release an embedded essential oil, maintaining an active concentration at the surface where microbial contamination is highest [168]. In another study, edible coating based on citrus oils from various fruits, when applied to strawberries, markedly prolonged their shelf life during cold storage (2 °C), preventing visible spoilage for 18 days in treated samples, in contrast to rapid degradation observed in untreated fruit [224]. This ~1.5× shelf-life extension without the use of fungicides illustrates the potent antifungal activity of natural plant oils. In other whole fruits like bananas, natural edible coatings like chitosan-tween have been employed to delay ripening. These coatings effectively slow respiration rates and color changes, thereby extending shelf life under ambient storage conditions [240]. Other available coatings include κ-carrageenan, a bio-coating capable of reducing postharvest ripening and moisture loss, consequently extending freshness [238]. Edible coatings formulated from food-grade polymers and natural additives (e.g., soy protein isolates or whey protein with sunflower oil) are also able to extend fruits shelf life compared to uncoated controls [241]. Another example is a composite chitosan-beeswax coating applied to fresh-cut pineapple, which significantly slowed microbial growth and preserved quality up to 12 days vs. ~7 days for controls [225]. Moreover, several studies have shown coatings with natural agents effectively inhibit surface growth of bacteria and molds on produce and deli products [242]. An additional benefit is that such bio-coatings can reduce reliance on plastic packaging by providing some protection themselves [243], aligning with sustainability trends.

Regarding active packaging, this approach involves incorporating the bioactive agents into packaging materials, allowing for their gradual release into the food environment during storage. Active packaging systems are designed not only to contain the product but also to interact with it in a functional manner, extending shelf life and maintaining microbial stability without direct incorporation of antimicrobials into the food matrix [244]. This form is particularly useful for bakery products, cheeses, and fresh produce. Examples of active packaging include sachets that release essential oil vapors, such as lemongrass oil used for bread preservation, and antimicrobial coatings applied to the inner surface of packaging films. For instance, bio-based films coated with compounds like nisin or oregano oil have been developed for products such as cheese [219]. Similarly, cinnamon oil in vapor phase extended bread’s shelf life from 4 days to 6 days even under warm conditions (37 °C) by continuously inhibiting fungal growth [212]. In another study, paper-based packaging infused with oregano oil and tea polyphenols significantly delayed fungal growth and prolonged the shelf life of strawberries [228]. An active packaging system combining grape seed extract (80 mg/m^2^), cinnamaldehyde (200 mg/m^2^), and nisin (60 mg/m^2^) significantly reduced psychotropics and anaerobic bacterial counts by 1–2 log CFU/g and decreased *L. monocytogenes*, *S. aureus*, and *C. jejuni* by approximately 4.7, 0.81, and 3.1 log CFU/g, respectively, in beef stored at refrigeration temperature for 28 days, being *C. jejuni* was undetectable after 21 days of storage [184]. A notable practical advantage of this approach is that it does not require the preservative to be added directly to the food product. Instead, the active compounds may volatilize or migrate in controlled amounts, which can be perceived by consumers as a more natural form of preservation.

## 5. Challenges and Safety Considerations

**Figure 4:**
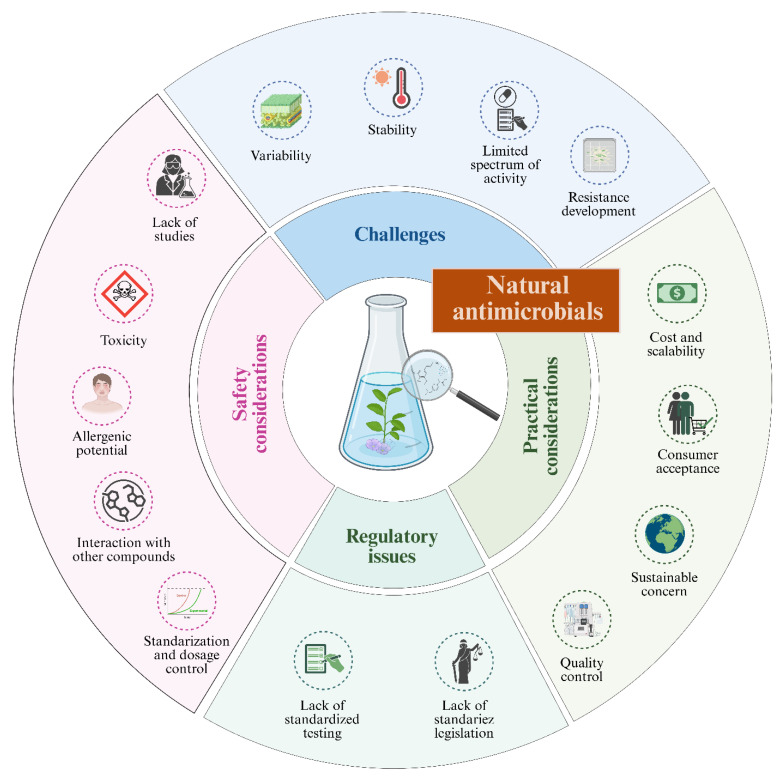
Limitations and safety considerations of natural antimicrobials.

Despite the wide variety of applications reported in the literature, natural antimicrobials face several technical and regulatory challenges that make their incorporation into new food matrices complex [245]. The first challenge is the variability in the composition of the extracts and, therefore, their efficacy (Figure 4). This is because, unlike compounds produced by chemical synthesis, natural extracts show fluctuations in their phytochemical profile depending on genotype, soil, climate conditions, harvest time, and the extraction methods used [246,247]. This plant inherent variability has a direct impact in production reproducibility and preservative efficacy [248], making difficult to reproduce plant extracts composition and antimicrobial effect, which represents a significant barrier to the standardization and industrial validation of natural antimicrobials preservatives.

In addition to compositional variability, the food matrix exerts a strong influence on antimicrobial performance. Interactions with macromolecules such as proteins and lipids can sequester active compounds, decreasing their bioavailability and reducing antimicrobial action relative to in vitro conditions [21,249]. For example, several studies have demonstrated that in fat-rich foods, numerous bioactive compounds interact with lipids, thereby decreasing their bioavailability and, consequently, their antimicrobial efficacy [250]. Other studies have shown that some phenolic compounds can form complexes with proteins, decreasing their ability to interact with microbial membranes [251]. Similarly, some phenolic compounds can form complexes with proteins, further restricting their interaction with microbial membranes. Physical and chemical factors such as pH, water activity, and viscosity also modulate antimicrobial efficacy, necessitating product-specific evaluations when selecting and dosing natural antimicrobials, making necessary a case-by-case evaluation [252].

Another significant technical limitation is compound instability under processing and storage conditions. Many plant-derived antimicrobials are sensitive to heat, light, oxygen, or pH changes, which can accelerate degradation and reduce activity [253,254,255]. For example, terpenes present in essential oils, such as thymol or carvacrol, can degrade at moderate temperatures. This degradation leads to a progressive reduction in antimicrobial activity, which requires the optimization of manufacturing and preservation processes [256].

To avoid these problems, numerous formulation strategies have been proposed. Approaches such as microencapsulation, nanoemulsions, liposomal carriers, immobilization onto solid matrices, or incorporation into edible coatings can enhance stability, protect bioactive compounds from environmental stressors, and allow controlled or sustained release [21,92]. These techniques facilitate controlled and sustained release, which improves its efficacy and prolongs its effect during storage or consumption of the product [257]. However, these technical solutions increase formulation costs and can interfere with the sensory properties of the food, so further scientific studies are needed.

Sensory acceptance is another critical factor influencing the applicability of natural additives, as many of these extracts or compounds possess intense aromas and flavors, along with low solubility, which may result in consumer rejection [21]. Such is the case with extracts derived from oregano, cloves, or cinnamon, which are added to some baked goods [258]. However, in some meat products, the same extracts have been shown to increase consumer acceptability [259]. In this regard, the use of synergistic combinations of natural antimicrobials at lower doses, or their controlled release, has been a promising way to maintain efficacy without compromising sensory quality [260]. Conducting further studies that not only examine combinations of different natural antimicrobials but also assess their integration with other preservation technologies is essential, as no single preservation method is fully effective on its own, especially when natural agents with milder action are used. For this reason, in many cases, a combination of multiple preservation factors is used, a strategy known as hurdle technology, which seeks synergistic action to optimize antimicrobial efficacy without compromising product quality [261].

Another crucial factor influencing consumer acceptance of these products is the way consumers interact with them. In general, products perceived as natural tend to enjoy greater public approval, despite limited consumer knowledge regarding the properties of the compounds incorporated into the matrix. This can lead to consumer confusion or deception if labeling is not correct. Consequently, it is crucial to strengthen consumer education regarding these matters and to promote effective knowledge transfer from industry stakeholders, to mitigate the risk of diminished consumer trust [262].

From a microbiological perspective, microorganisms can enter at any stage of production, and their behavior is less predictable than chemical contamination. While chemical contaminants can accumulate and cause long-term effects, microbial pathogens typically cause illness quickly [263]. Moreover, not all natural compounds or extracts have the same antimicrobial potential, and their spectrums of action vary widely. It has been shown that many of these compounds have limited antimicrobial activity, being effective only against Gram-positive or Gram-negative bacteria, but not both [264,265]. Likewise, less effectiveness has been observed against molds and yeasts [266]. Thus, ensuring the microbiological safety of food often requires not only the judicious selection of such compounds but also their integration with complementary preservation techniques.

In addition to technological limitations, safety and regulatory factors are central to the practical application of plant-derived antimicrobials. Fundamental to safety assessment is the recognition that traditional use of a plant does not automatically equate to safety of its concentrated extracts or isolated compounds. Toxicological evaluation must consider acute and chronic effects, dose–response relationships, and interactions within complex matrices, which can alter bioavailability and metabolic fate [267,268,269,270]. For many bioactive compounds, standardized toxicological reference values such as Acceptable Daily Intake (ADI) or No Observed Adverse Effect Level (NOAEL) are unavailable, especially when dealing with complex extracts containing multiple bioactive constituents [271]. This gap complicates risk assessment, as extracts may exert synergistic or cumulative effects that are not predictable from individual components. For example, allicin from garlic and eugenol from clove have demonstrated cytotoxicity in some in vitro systems at high concentrations, and certain alkaloids or glycosides can possess mutagenic or genotoxic potential [272]. These findings underscore the need for comprehensive toxicology studies that go beyond single-constituent testing. Furthermore, prolonged exposure to sublethal levels could have adverse effects that are not yet fully understood.

Another safety concern relates to emergence of microbial resistance. Although less extensively studied than antibiotic resistance, prolonged use of some natural antimicrobials such as bacteriocins has been shown to exert selective pressure on microbial populations, potentially favoring resistant strains [273]. Ongoing monitoring and evaluation of long-term use effects is therefore essential.

In any case, the results obtained from toxicological analyses must comply with the requirements established by current regulatory frameworks. These requirements vary by country; however, most regulatory authorities dictate rigorous testing for efficacy, safety, and stability prior to the approval of a new food ingredient. In the United States, the FDA oversees approval of food additives and may grant status through mechanisms such as Generally Recognized as Safe (GRAS) when sufficient evidence supports safety for intended use. Traditional foods or ingredients consumed prior to 1994 may be exempt from premarket approval under the Dietary Supplement Health and Education Act (DSHEA), but this exemption does not automatically apply to concentrated extracts intended for antimicrobial function [271]. In the European Union, The European Food Safety Authority (EFSA) evaluates botanicals and novel ingredients under specific risk-assessment frameworks, requiring detailed safety and exposure data before approval. Ingredients with a history of traditional use prior to 15 May 1997, in accordance with EFSA criteria, may be exempt from some premarket requirements, like the provisions in the United States under DSHEA [274]. However, natural extracts are often not registered as additives, but rather as functional ingredients, which complicates their use as declared antimicrobial agents [275]. Additionally, most available studies evaluate the antimicrobial capacity of an extract, not of specific compounds, making their food safety studies and legislative approval more complex [254,255]. Legal categorization can vary depending on the method of extraction, purity, or even the country of origin, creating regulatory uncertainty for manufacturers [275]. This lack of harmonization hampers their global application and can hinder their industrial adoption.

Finally, from a sustainability perspective, large-scale production of plant-derived antimicrobials can present environmental and resource challenges. Intensive use of land, water, and energy, as well as solvent use and waste generation in extraction processes, can diminish environmental benefits unless green extraction technologies are implemented [276,277,278]. It is also essential that the extraction of target compounds does not compete with primary food resources [279]. Therefore, although promising, these approaches still face technical and economic barriers to wide adoption.

In summary, the use of natural antimicrobials in food products poses multiple challenges that go beyond their ability to inhibit microorganisms. Compositional variability, the influence of the food matrix, stability during processing, sensory compatibility, antimicrobial spectrum, toxicological safety, regulatory barriers, and sustainability are all interdependent factors that must be carefully evaluated. Despite these difficulties, the development of innovative solutions and the integration of multidisciplinary knowledge pave the way for the safe and effective application of these compounds in the modern food industry.

## 6. Conclusions

Phytochemicals isolated from plants represent a potential alternative to synthetic additives in the food industry. Among these, essential oils, phenolic compounds, alkaloids, terpenes, and saponins have shown notable activity against foodborne pathogens. However, although plant-derived antimicrobials hold significant potential as environmentally friendly alternatives to synthetic preservatives, their practical application in the food industry still faces several key challenges, including standardization, matrix-dependent efficacy, toxicological evaluation, and regulatory approval. Addressing these challenges will be critical to developing effective natural preservatives that maintain microbiological safety while preserving the organoleptic and nutritional quality of foods. Future research should aim to deepen our understanding of the mechanisms of action of individual phytochemicals and their interactions within complex food matrices. Advances in formulation science, such as encapsulation, controlled-release systems, and combination strategies, can enhance stability, efficacy, and sensory compatibility. Additionally, integrating predictive approaches like metabolomics and computational modeling can help anticipate interactions and optimize applications. Alongside thorough toxicological and regulatory evaluation, these strategies will facilitate the practical implementation of plant-derived antimicrobials in commercial foods, supporting more sustainable and safe preservation solutions while opening avenues for innovative, application-oriented research in the field.

## Figures and Tables

**Table 1:** A summary of antimicrobial studies that make use of plant extracts.

Raw Material	Extraction	Microorganism	Assay Type	Results	Ref.
**Polyphenols**					
*Sida alba* L. (L, S)	SE: H_2_O:Ac (80%, *v*/*v*)	Co-trimoxazol RBS	MIC	0.0125–0.05 mg/mL; MBC: 0.025–0.2 mg/mL	[42]
*Foeniculum vulgare*	HD: 360 min; 100 °C	*E. coli*, *S. tiphy*, *Bacillus cereus*, *S. aureus*, *A. flavus*, *C. albicans*		0.0125, 0.015, 0.0125, 0.01, 0.01, 0.01 mg/mL	[43]
*Gratophyllum grandulosum* (L)	MA (chrysoeriol and lutein derivatives): RT, EtOAc	*Vibrio cholerae* SG24, CO6, NB2, PC2		0.004–0.064 mg/mL	[44]
*Macaranga tanarius* (F)	MA (propolin D): EtOH, 3 days	*S. aureus*, *S. epidermis*, *C. albicans*		0.010–0.050 mg/mL	[45]
*Trianthema decandra* (AP)	SE (quercetin): petroleum eter, 40–60 °C	*Pseudomonas aeruginosa*		0.039 mg/mL	[46]
*Dracocephalum moldavica* (L)	MA (tilianin, luteolin): EtOH 60%, 60 °C, 2 h	*S. aureus*		50–90 mg/mL	[47]
*Centaurea damascena* (AP)	MA (fokienol, thymol, α-terpineol): MeOH	*E. coli*, *K. pneumonia*, *Micrococcus lutes*		0.06–1.1 mg/mL	[48]
*Sophora flavences* (R)	CE (sophoraflavanone G, kurarinone)	*MRSA*		0.0039–0.0078 mg/mL	[49]
*Pomegranate* (P)	MA (flavonoids): EtOH 96%	*Shigella flexineri*, *Salmonella typhi*, *S. aureus*		25–100 mg/mL	[50]
*Alchornea laxiflora* (L)	MA (flavonoids): MetOH/H_2_O 3:1	*B. polymyxa*		0.78–1.56 mg/mL	[51]
*Croton macrostachyus* (R)	SE: H_2_O:MetOH (80%, *v*/*v*)	*S. aureus*, *S. pneumonia*, *E. coli*, *K. pneumonia*	IZ	5.8, 6.2, 9, and 6.0 mm (100 μg)	[52]
*Mentha* sp., *Tinospora cordifolia*, *Cymbopogon citratus*, *Foeniculum vulgare*, *Cassia absus*, *Camellia sinensis*, *Trachyspermum ammi*	L, S//UAE: 10 g plant, 200 mL MetOH (80%), 1 h, 37 kHz, 50 °C	*E. coli*, *Salmonella enterica*, *S. aureus*, *B. cereus*		7.3–19.3 mm (25–400 μL/mL)	[53]
**Terpenoids**					
*Syzygium samarangense* (L)	HD (EO): 300 min; 100 °C//SFE (EO): CO_2_; 180 min; 40 °C; 148 atm	*S. aureus*, *E. faecalis*, *E. coli*, *C. albicans*		0.00375–0.0075 mg/mL//0.00585–0.0137 mg/mL	[54]
*Syzygium malaccense* (L)				0.00375–0.015 mg/mL//0.00585–0.0117 mg/mL	
*Coriandrym sativum* (SE)	MAHD (EO): 100 °C; 1 atm; 10 min, 800 W and 60 min, 500 W	*S. aureus*, *P. aeruginosa*, *A. niger*, *C. albicans*	MIC	16, 128, 2, and 16 mg/mL	[55]
	HD (EO): 240 min; 100 °C			32, 64, 4, and 8 mg/mL	
*Cinnamomum camphora*	CE: (EO: terpinene-4-ol)	*S. agalactiae*		0.098 mg/mL	[56]
*Andrographis paniculata*	CE (andrographolide)	*MRSA*, *E. coli*, *B. subtilis*, *Streptococcus pneumoniae*		0.1–0.25 mg/mL	[57]
*Mikania glomerata*	MA (kaurenoic acid): MeOH/H_2_O 9:1 *v*/*v*	*P. micra*		0.00312 mg/mL	[58]
*Rabdosia rubescens*	CE (oridonin)	*MRSA*		0.064–0.128 mg/mL	[59]
*Azadirachta indica* (L)	SE (β-D-mannofuranoside, O-geranyl):hexane, EtOH, W, 40 °C	*E. coli*, *S. aureus*, *Parvimonas aeruginosa*, *C. albican*, *Aspergillus parasiticus*		0.05–0.2 mg/mL	[60]
*Artemisia annua* L. (L)	HD (EO): 135 min; 100 °C	*B. subtilis*, *B. cereus*, *S. aureus*, *E. coli*, *K. pneumoniae*, *Salmonella* sp.	IZ	37, 11, 23, 75, 22, and 16 mm	[61]
*Pelargonium graveolens*, *Citrus bergamia*, *Cinnamomum zeylanicum*, *Lavandula angustifolia*	CE (EO: linalol)	*S. aureus*, *P. aeruginosa*, *C. albicans*		9–50 mm (10 μL)	[62]
**Alkaloids**					
*Cinchona officinalis*	CE	*C. albicans*	MIC	0.25 mg/mL	[63]
*Amaryllis belladonna* (F)	PE (amarbellisine, lycorine, pancracine, vittatine, hippeastrine): EtOH	*S. aureus*, *E. coli*, *P. aeroginosea*, *C. albicans*		0.125, 0.024, 0.063 mg/mL	[64]
*Phellodendron amurense* (H)	SE: W:EtOH 80%, *v*/*v*	*E. faecium*, *S. aureus*, *S. pyogenes*, *E. coli*, *K. pneumonia*, *P. aeruginosa*		7.353 mg/mL	[65]
*Toddalia asiatica* (R)	MA (chelerythrine): MetOH, 7 days, 3 times	*S. aureus*		0.156 mg/mL	[66]
*Toddalia asiatica* (R)	MA (chelerythrine): MetOH, 7 days, 3 times	*S. aureus*	IZ	19.97 mm	[66]
**Sulfur-containing compounds**					
*Allium sativum*	CE (allicin)	*S. aureus*, *E. coli*, *P. aeruginosa*, *K. pneumonia*	LD_50_	0.012, 0.015, 0.015, 0.008 mg/mL	[67]
*Allium sativum*	CE (allicin)	*S. aureus*, *P. aeruginosa*	CPSI	50 and 70%	[68]
*Petiveria alliacea*	CE (diallyl disulfide)	*B. cereus*, *S. aureus*, *E. coli*	MIC	0.0003, 0.002 mg/mL	[69]
**Saponins**					
*Dioscorea nipponica* (R)	MA (dioscin): MeOH, 5 L, 3 kg sample	*Trichosporon beigelii*, *Malassezia furfur*, *C. albicans*, *Candida parapsilosis*	MIC	0.01–0.02 mg/mL	[70]
*Glycyrrhiza glabra* (R)	MA (glycyrrhizic): EtOH 80%, 72 h, RT	*Streptococcus* spp., *Actinomyces viscosus*, *Enterococcus faecalis*, *S. aureus*, *E. coli*		12.5–50 mg/mL	[71]
*Melanthera elliptica* (AP)	MA (GLU): MeOH, 3 × 20 L, 72 h	*E. coli*, *S. aureus*, *S. flexneri*		0.512, 0.128, 0.256 mg/mL	[72]
*Chenopodium quinoa* (H)	UAE (N.S): EtOh, 1 h, 30 min	*S.aureus*, *S.epidermidis*		0.0625 mg/mL	[73]
*Camellia oleifera* (SE)	UAE (camelliagenin): 1 h, 60 °C, 300 W, HCl 2%	*E. coli*, *S. aureus*	IC_50_	0.05, 0.08 mg/mL	[74]
**Others**					
*Nigella sativa* (SE)	SE (polypetide: thionin):10 % acetic acid, 1:7, *w*/*v*	*B. subtilis*, *C. albicans*	MIC	0.0163–0.0325 mg/mL	[75]
*Pingyin rose* (F)	MA (organic acids): EtOH 95%, 90 °C, 2 h	*S. aureus*		0.19–0.39 mg/mL	[76]
*Abutilon theophrasti*	MA (hibicuslide C): MeOH 80%	*P. aeruginosa*		0.005–0.01 mg/mL	[77]
*Moringa oleifera* (S)	CE (glucosinolate)	*B. cereus*, *Cronobacter sakazakii*	CPSI	0.28–0.50%	[78]
*Nicotiana* spp. (L)	CE (coumarin: daphnetin)	*Ralstonia solanacearum*	N.S.	N.S.	[79]

Abbreviations. Extraction: E: extract; EO: essential oil; MA: maceration; UAE: ultrasound assisted extraction; SE: solvent extraction; MetOH: methanol; MAHD: Microwave assisted hydrodistillation; HD: hydrodistillation; SD: steam distillation; SFE: supercritical fluid extraction; PE: percolation; EtOH: ethanol; W: water; CE: commercial extract. Plant material: L: leaves; S: stems; P: peel; R: roots; F: fruits; SE: seeds; AP: aerial parts; H: husk. Assay: MIC: minimum inhibitory concentration; MBC: minimum bactericidal concentration; IZ: inhibition zone; CPSI: cumulative percentage of strains inhibited. Microorganisms: RSB: resistant bacterial strains; MRSA: methicillin-resistant *Staphylococcus aureus*. Others: N.S.: not specified; GLU: 3-O-β-d-glucuronopyranosyl-oleanolic acid.

**Table 2:** Maximum allowed levels and food applications of synthetic preservatives according to European Union regulations [157,158].

Preservative	Structure	Limits	Allowed Uses	Targets	Restrictions
Potassium nitrite, sodium nitrite, sodium nitrate, potassium nitrate (E249–E252)	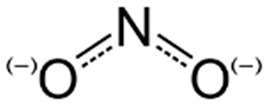	50–300 mg/kg	Processed meats, cured meats	*Clostridium botulinum*, bacteria	Strict limits due to nitrosamine formation; forbidden in infant products
Benzoic acid, sodium benzoate, potassium benzoate, calcium benzoate (E210–E213)	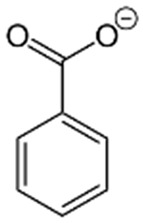	150–1000 mg/kg	Soft drinks, fruit juices, pickles, condiments	Yeasts, some bacteria	Can form benzene in presence of vitamin C under specific conditions
Sorbic acid, potassium sorbate, calcium sorbate (E200–E203)	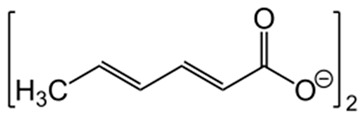	300–2000 mg/kg	Bakery products, cheeses, beverages, sauces	Molds, yeasts	Not allowed in infant foods; efficacy increases at low pH
Propionic acid, sodium propionate, calcium propionate, potassium propionate (E280–E283)	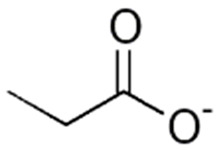	2000–3000 mg/kg	Breads, pastries	Molds	Typically limited to bakery; sensory changes at high doses
Sulphur dioxide, sodium sulfite, sodium hydrogen sulfite, sodium metabisulphite, potassium metabisulphite, calcium sulfite, calcium hydrogen sulfite, potassium hydrogen sulfite (E220–E228)	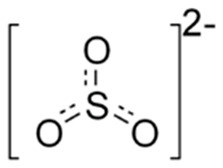	10–2000 mg/kg as (as SO_2_)	Wine, processed potatoes, cereals, snacks	Molds, yeasts, bacteria	Must not exceed total SO_2_ limits; not allowed in infant foods; may trigger asthma or hypersensitivity
Natamycin (E235)	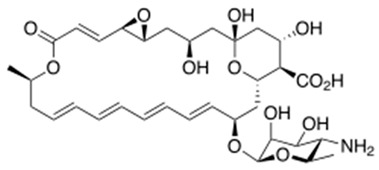	Surface application only (1–10 mg/dm^2^)	Cheeses, cured meat	Fungi	Penetration into food must not exceed 5 mm

**Table 3:** Plant-derived extracts as antimicrobial agents for food industry applications.

Source	Extraction	Product	Treatment	Inhibition	Ref.
**Incorporation into meat products**					
Basil	EO (HD, 100 °C, 8–10 h): nd	Smoke fermented sausages	9.0 μL/mL//21 days, 4 °C	*Penicillium carneum*, *Penicillium polonicum*	[177]
Basil, rosemary	EO (CE): nd	Chicken	2.5, 5.0 mg/mL//16 days, 4–18 °C	*Salmonella enteritidis*	[174]
Chestnuts	E (EtOH, 60 °C, 24 h): polyphenols	Chicken	1 mg/mL//4 days, 4 °C	*Campylobacter jejuni*	[172]
Chestnuts	E (W*, 25 °C, 90 min): polyphenols	Beef patties	1000 mg/kg//18 days, 2 °C	*Pseudomonas* spp., LAC	[178]
Cinnamon	CE: cinnamaldehyde, eugenol	Beef	0.003–0.8%//2 days, 35 °C	*Pseudomonas putida*	[179]
Cinnamon, oregano	EO (CE): nd	Roast duck	0.60%, 0.15%//21 days, 2 °C	TVC, *Enterobacteriaceae*, LAC	[180]
Cinnamon, cloves	EO (CE): nd	Ground beef	5% + 5%//60 days, −18 °C//7 days, 0–8 °C	*Listeria monocytogenes*	[181]
Cranberry	E (EtOH, 70 °C, 2 h): polyphenols	Minced pork	2.5%//6 days, 4 °C	*Staphylococcus aureus*, *L. monocytogenes*, *S. enteritidis*, *Escherichia coli*	[182]
Grapefruit	CE (Citricidal^®^): nd	Sous-vide chicken	200 ppm//9.5 h, 19–25 °C	*Clostridium perfringens*	[183]
Grapefruit	CE: nd	Beef	80 mg/m^2^ + cinnamaldehyde + nisin//28 days, 4 °C	TPB, TAB	[184]
Grapefruit	CE: polyphenols	Hamburgers, salami	2.5%//15 days, 4 °C	*S. aureus*, *L. monocytogenes*, *Pseudomonas aeruginosa*, *E. coli*	[185]
Grapefruit	CE: nd	Türkiye frankfurters	0.5%//28 days, 4 °C	*E. coli*, *L. monocytogenes*, *Salmonella typhimurium*	[186]
Lemon	EO (HD, 100 °C, 3 h): β-PIN, LIM, LIN, α-TERP, LIN-Ac, GER-Ac, NER, NER-Ac, FAR	Beef	0.312 mg/g//10 days, 4 °C	*L. monocytogenes*	[187]
Oregano	EO (CE): CAR, THY, p-CYM, β-CAR, γ-TER, α-HUM, α-PIN	Wildebeest	1%//3 days, 3 °C	TVC, LAC, coliform counts	[188]
Oregano	EO (nd): CAR, p-CYM, γ-TER	Minced beef	0.2% + 0.5% caprylic acid//10 days, 3 °C	*L. monocytogenes*, LAC, psychrotrophic bacteria	[189]
Oregano	EO (CE): CAR, p-CYM, THY	Dried meat	3 mL for filter paper//nd	*E. coli*, *S. enteritidis*	[190]
Pear	SE (W, RT, 10 min): polyphenols	Beef burger	0.5%//8 days, 4 °C	*Enterobacteriaceae*, *Pseudomonas* spp.	[191]
Pineapple	SE (EtOH, RT, 25 min): polyphenols	Beef	5%//5 days, 4 °C	*Pseudomonas* spp.	[192]
Pomegranate	EO (W, 40 °C, 24 h): CAR, THY, CIS-GER, CAR, GER-D, CAM, α-TERP, TER-4-OL, LIM, CIS-β-TERP, p-CYM, γ-TER	Beef	0.5–1%//21 days, 4 °C	*Pseudomonas* spp., LAC, *L. monocytogenes*	[193]
Rosemary	EO (CE): nd	Chicken	5 mg/mL//24 h, 18 °C	*S. enteritidis*	[174]
Rosemary	E (EtOH, 30 min, RT): CIN, α-PIN, CAM, CAMP, CAR	Beef	45%//9 days, 4 °C	*E. coli*, *S. enteritidis*, *L. monocytogenes*	[194]
Sage	EO (EtOH, Soxhlet): polyphenols	Chicken	0.1%//9 months, −18 °C	TAB, TPB, coliforms, *Enterobacteriaceae*	[195]
Sage	EO (HD, 3 h): terpenes, β-PIN, β-TH, CAM, α-HUM	Sous-vide beef	0.625%//28 days, 2–8 °C	*L. monocytogenes*	[196]
*Salvia officinalis*, *Schinus molle*	EO (HD, 100 °C, 3 h): CIN, β-TH, α-TH, BO//α-PHE, β-PIN, p-CYM, α-PIN	Mince beef	1.5 and 2%//15 days, 4–7 °C	*Salmonella* sp.	[197]
Thyme	EO (CE): CARY, CM, 4-CAR, α-PIN, LIN, EPO, CPH, β-PIN, BIC, BOR	Meat sausage	0.95% + 1% powdered juice//15 days, 4 °C	*S. aureus*, *E. coli*	[198]
Thyme	EO (CE): THY, p-CYM, CARY, CPH, α-PIN	Chicken breast	1% + juice + 0.5% *Yucca schidigera*//8 h, 22 °C	*Salmonella enterica*	[173]
Thyme	EO (CE): THY, p-CYM, LIN, γ-TER, α-PIN, CAR	Hamburgers	1%//14 days, 4 °C	*S. aureus*, *E. coli*, *L. monocytogenes*, *S. typhimurium*	[199]
Turmeric	CE: nd	Chicken	3%//14 days, 4 °C	TAB, coliforms	[200]
**Incorporation into horticultural products**					
Cinnamon	EO (CE): nd	Orange juice	650 mg/mL//2 days, 25 °C	*Saccharomyces cerevisiae*	[201]
Cinnamon, clove	EO (CE): nd	Strawberry jams	0.65 mg/g + 0.55 mg/g//nd	*Aspergillus flavus*, *Aspergillus niger*, *Penicillium expansum*	[202]
Cinnamon, thyme	EO (CE): nd	Tomato juice	0.625 μL/mL//24 h, 37 °C	*L. monocytogenes*	[203]
Citrus	EO (CE): LIN, LIM, α-TERP, α-PIN	Orange juice	0.5 μL/mL	*Lactobacillus brevis*, *Leuconostoc mesenteroides*	[204]
Eucalyptus	EO (CE): nd	Orange juice	0.8–4 µL/mL//6 days, 4 °C	*S. cerevisiae*	[205]
Melissa	EO (CE): nd	Watermelon juice	2 µL/mL//7 days, 4 °C	*L. monocytogenes*	[206]
Mint	EO (HD, 6 h): CAR	Apple juice	0.1%//41 days, 4–20 °C	*Zygosaccharomyces bailii*, *Zygosaccharomyces rouxii*	[207]
Oregano, thyme	EO (CE): CAR, THY	Wine	0.1%//24 h, 37 °C	*E. coli*	[208]
*Pistacia lentiscus*, *Fortunella margarita*	EO (HD): α-PIN, β-PIN, LIM	Fruit juice and ice cream	0.2% + 0.006%//24 h, −23–4 °C	*E. coli*, *L. monocytogenes*, *Pseudomonas fragi*, *A. niger*, *S. cerevisiae*	[209]
Thyme	EO (HD): nd	Pomegranate juice	0.125%//245 days, 5–20 °C	Aerobic mesophilic bacteria, *Streptococcus thermophilus*, molds, yeasts	[210]
Vanillin	EO (CE)	Coconut water	0.1%//24 h, 37 °C	*S. typhimurium*	[211]
**Incorporation into bakery products**					
Cinnamon	EO (CE): polyphenols	Bread	0.5 g//3 days, RT	TVC, yeasts, molds	[212]
Cinnamon	EO (CE): nd	Bread	1.3%//30 °C	*Rhizopus stolonifera*, *Aspergillus flavus*	[213]
Citrus	EO (CE): nd	Cake	2.5%//4 weeks, 4 °C	*Aspergillus* spp., *Fusarium* spp., *Penicillium* sp.	[214]
Clove	EO (CE): nd	Wheat flour	Nd//21 days, RT	*Penicillium oxalicum*, *A. flavus*	[215]
Clove	EO (HD): nd	Cake	800 ppm//28 days, RT	TVC, yeasts, molds	[216]
Clove, orange	EO (HD): EUG	Bread	1–2%//21 days, RT	Yeasts, molds	[217]
Coriander	EO (HD): CAM, α-PIN, LIM	Cake	0.15%//60 days, RT	Yeast, molds	[218]
Lemongrass	EO (CE): LIM, LIN, CAMP, citral, EUG	Bread	125 to 4000 μL/L_air_//21 days, RT	*P. expansum*	[219]
Oregano	EO (HD): CAR, THY	Bread	200 mg/kg//20 days, RT	*Aspergillus* spp.	[220]
Thyme	EO (CE): CAM, α-PIN, LIM, LIN, CAR, THY	Cake	0.5 mg/mL//30 days, RT	*Candida albicans*, *Enterococcus faecium*, *Enterococcus hirae*, *E. coli*, *Salmonella choleraesuis*, *S. aureus*, *S. typhimurium*, *P. aeruginosa*, *A. niger*	[221]
**Active packaging**					
Aloe vera, ginger, garlic	SE: polyphenols	Guava	Up to 20%//15 days, 25 °C	Molds, yeasts	[222]
Apricot	SE (HClO, RT, 15 min): polyphenols	Fresh-cut vegetables	9%//15 days, 4 °C	*S. aureus*, *S. typhimurium*	[223]
Cinnamon	CE: cinnamaldehyde, eugenol	Pineapple	0.5% + 2% chitosan//15 days, 5 °C	Molds, yeasts, *Salmonella* spp., *E. coli*	[180]
Citrus	EO (CE): LIM	Strawberries	2%//18 days, 2 °C	*S. aureus*, *P. aeruginosa*, *Bacillus subtilis*, *S. typhimurium*, *A. niger*, *A. flavus*, *S. cerevisiae*, *Candida lypolitica*	[224]
Lemongrass	EO (CE): nd	Pineapple	0.3%//16 days, 10 °C	Molds, yeasts	[225]
Mulberry	E (W, RT, 16 h): polyphenols	Capsicum	10%//15 days, RT	*P. aeruginosa*, *Bacillus cereus*	[226]
Mustard	EO (CE): nd	Bread	1%//3 days, RT	*Penicillium roqueforti*, *Penicillium commune*, *Endomyces fibuliger*	[227]
Oregano	EO: polyphenols	Strawberry	40 g/m^2^//8 days, 25 °C	TVC	[228]
*Satureja* spp.	EO (HD, 100 °C, 3 h): nd	Strawberries	300 µL/L//RT	*Rhizopus stolonifer*, *Penicillium digitatum*, *A. niger*, *Botrytis cinerea*	[229]
Tea	CE: polyphenols	Meat	30%//2 days, 37 °C	TVC	[230]
Thyme	EO (CE): THY	Blueberry	1 mg/mL//10 days, 7 °C	*Botrytis cinerea*	[231]
*Zataria multiflora*	EO (HD): DIH, POS	Chicken	1%//16 days, 4 °C	TVC, LAC, psychotropic bacteria, *Pseudomonas* spp.	[232]

Abbreviations. Extraction: EO: essential oil; HD: hydrodistillation; E: extract; EtOH: ethanol; W*: acidified water; CE: commercial extract; nd: not determined; SE: solvent extraction; W: water; HClO: hypochlorite solution 0.005% (*w*/*v*). Essential oils: β-PIN: β-pinene; LIM: limonene; LIN: linalool; α-TERP: α-terpineol; LIN-Ac: linalyl acetate; GER-Ac: geranyl acetate; NER: nerolidol; NER-Ac: neryl acetate; FAR: farnesol; CAR: carvacrol; THY: thymol; p-CYM: ρ-cymene; β-CAR: β-caryophyllene; γ-TER: γ-terpinene; α-HUM: α-humulene; α-PIN: α-pinene; CIS-GER: cis-geraniol; CARY: caryophyllene; GER-D: germacrene-D; CAM: camphor; TER-4-OL: terpinen-4-ol; CIS-β-TERP: terpineol cis-beta; CIN: 1,8-cineole; CAMP: camphen; β-TH: β-thujene; α-TH: α -thujene; BO: borneol; PHE: phellandrene, CPH: camphor; CM: O-cymol; 4-CAR: 4-carene; EPO: cis-Z-α-bisabolene epoxide; BIC: 3-(acetylmethyl), bicyclo [7.2.0]undec-4-ene, 4,11,11-trimethyl-8-methylene-,[1R-(1R*,4Z,9S*)]; BOR: isoborneol; EUG: eugenol; DIH: dihydrochrysin; POS: pinostrobin. Inhibition: LAC: lactic acid bacteria; TVC: total viable count; TAB: total aerobic bacteria; TPB: total psychrotrophic bacteria.

## Data Availability

No new data were created or analyzed in this study. Data sharing is not applicable to this article.

## Abbreviations

The following abbreviations are used in this manuscript:

BHA	Butylated hydroxyanisole
BHT	Butylated hydroxytoluene
HD	Hydrodistillation
SD	Steam distillation
MAE	Microwave assisted extraction
SFE	Supercritical fluid extraction
MAHD	Microwave-assisted hydrodistillation
TPC	Total phenolic content
MBC	Minimum bactericidal concentration
MIC	Minimum inhibitory concentration
IZ	Inhibition zone
VOCs	Volatile organic compounds
EOs	Essential oils
GRAS	Generally recognized as safe
EPS	Extracellular polysaccharide
MOAs	Modes of action
MRSA	Methicillin-resistant *Staphylococcus aureus*
MscL	Large-conductance mechanosensitive channel
QS	Quorum sensing
PAβN	Phenylalanine-arginine beta-naphthylamide
ADI	Acceptable daily intake
NOEL	No observed adverse effect level
DSHEA	Dietary Supplement Health and Education Act
EFSA	European Food Safety Authority
